# Adaptive patterns in the p53 protein sequence of the hypoxia- and cancer-tolerant blind mole rat *Spalax*

**DOI:** 10.1186/s12862-016-0743-8

**Published:** 2016-09-02

**Authors:** Vered Domankevich, Yarden Opatowsky, Assaf Malik, Abraham B. Korol, Zeev Frenkel, Irena Manov, Aaron Avivi, Imad Shams

**Affiliations:** 1 Institute of Evolution & Department of Evolutionary and Environmental Biology, University of Haifa, Haifa, Israel; 2 Mina and Everard Goodman Faculty of Life Sciences and Advanced Materials and Nanotechnology Institute, Bar-Ilan University, Ramat-Gan, Israel

**Keywords:** P53, Hypoxia, Oxidative stress, RPA70, TAD2, Convergent evolution, Cancer resistance, Longevity

## Abstract

**Background:**

The subterranean blind mole rat, *Spalax* (genus *Nannospalax*) endures extreme hypoxic conditions and fluctuations in oxygen levels that threaten DNA integrity. Nevertheless, *Spalax* is long-lived, does not develop spontaneous cancer, and exhibits an outstanding resistance to carcinogenesis in vivo, as well as anti-cancer capabilities in vitro. We hypothesized that adaptations to similar extreme environmental conditions involve common mechanisms for overcoming stress-induced DNA damage. Therefore, we aimed to identify shared features among species that are adapted to hypoxic stress in the sequence of the tumor-suppressor protein p53, a master regulator of the DNA-damage response (DDR).

**Results:**

We found that the sequences of p53 transactivation subdomain 2 (TAD2) and tetramerization and regulatory domains (TD and RD) are more similar among hypoxia-tolerant species than expected from phylogeny. Specific positions in these domains composed patterns that are more frequent in hypoxia-tolerant species and have proven to be good predictors of species’ classification into stress-related categories. Some of these positions, which are known to be involved in the interactions between p53 and critical DDR proteins, were identified as positively selected. By 3D modeling of p53 interactions with the coactivator p300 and the DNA repair protein RPA70, we demonstrated that, compared to humans, these substitutions potentially reduce the binding of these proteins to *Spalax* p53.

**Conclusions:**

We conclude that extreme hypoxic conditions may have led to convergent evolutionary adaptations of the DDR via TAD2 and TD/RD domains of p53.

**Electronic supplementary material:**

The online version of this article (doi:10.1186/s12862-016-0743-8) contains supplementary material, which is available to authorized users.

## Background

The blind mole rat of the genus *Nannospalax* (hereafter, *Spalax*) is a solitary subterranean mammal [1] that experiences extreme and abrupt fluctuations in O_2_/CO_2_levels. *Spalax* survives low-oxygen content (~7 % O_2_) in its natural underground habitat, and even lower (3 % O_2_) under laboratory conditions [2–4]. One of the greatest challenges faced by *Spalax* is repeated exposure to acute hypoxia followed by rapid re-oxygenation, which leads to oxidative stress [5]. Hypoxia and oxidative stress are two types of cellular stressors that place a great risk on cellular functions and genomic stability [6–8]. Importantly, the combination of these stressors seems to further enhance genomic instability compared to the separate effects of each stress type. Hypoxia depletes dNTPs and represses DNA repair pathways that are required to overcome replication-stress, while re-oxygenation induces replication-restart that coincides with reactive oxygen species (ROS)- induced oxidative DNA damage, at a time when the repair pathways have not yet recovered [9, 10].

Genomic instability underlies both cancer and aging [11]. Nevertheless, *Spalax* does not develop spontaneous tumors and does not show clear age-related phenotypic changes, despite its relatively long lifespan (~20 years in captivity) [12]. Moreover, *Spalax* displays an outstanding tolerance to chemically induced carcinogenesis in vivo, and its fibroblasts inhibit cancer growth in vitro [13]. Another long-lived (~30 years) subterranean rodent in which spontaneous cancer is an uncommon phenomenon, relative to similar-sized rodents, is the naked mole rat, *Heterocephalus glaber* (hereafter, *H. glaber*) [14, 15]*. H. glaber* is phylogenetically distant from *Spalax* (estimated divergence time is 77.9 million years) [16]. Yet, similar to *Spalax*, it shows adaptations to hypoxic stress [17] and in vitro ability to inhibit cancer cell growth [13]. Bearing in mind the well-known strong positive correlation between aging and cancer epidemiology in many metazoans, including humans [18], common features in *Spalax* and *H. glaber* deserve special attention. Such features may relate to evolutionary adaptations to common environmental conditions that might play a compensatory role in dealing with consecutive stress-induced DNA damage.

The subterranean habitat is not the only environment in which oxygen fluctuations may occur. Diving mammals experience rapid transitions from apnea to re-oxygenation [19], while hibernating mammals face reduced metabolism and fluctuations in blood flow and oxygen consumption [20–23]. “Metabolic shutdowns” happen also in desert mammals, such as the desert mouse [24] and the jerboa [25]. Evidence for oxidative stress or antioxidant adaptations were indeed found in *Spalax* [26], diving mammals [19, 27], hibernating mammals [22], and desert mammals [28]. Bats are another example of mammals showing antioxidant adaptations [29], as might be expected from the high metabolic stress these mammals experience during flight [30, 31]. Interestingly, bats’ lifespan increases with hibernation, body mass, and the occasional use of caves [32]. Their exceptional longevity is explained by multiple mechanisms for resisting oxidative damage [33] and is attributed to positively selected genes in DNA-damage-checkpoint pathways [34]. The above mentioned studies indicate that environmental conditions, which include extreme changes in oxygen supply, may require adaptations to cope with oxidative stress that are not restricted to antioxidant activity, but also include changes in the DNA-damage response (DDR) as a second line of defense.

Sophisticated DDR pathways constantly monitor genome integrity [35] and delay or stop cell-cycle progression at critical stages in response to unrepaired DNA damage, thereby preventing replication of the damaged DNA [36]. The tumor suppressor protein p53 interacts with multiple proteins participating in complex pathways of the DDR [37]. It plays an important role in cellular pathways that control genomic instability and is involved in critical defense and regulatory pathways such as cell-cycle arrest, senescence, and apoptosis [35]. The tight control by senescence and apoptosis prevents the uncontrolled proliferation of damaged cells; however, it may also deplete stem and progenitor cell pools, thus promoting tissue degeneration and aging [35]. This emphasizes the subtle role of p53 in regulating pathways related to cancer and aging [38]. *Spalax* has acquired an R174K amino acid substitution in the DNA-binding domain (DBD) of p53. Mutation at this position in human p53 leads to impaired induction of apoptotic and cell-cycle arrest genes and is known as a frequent mutation found in many human cancers [39, 40]. The fact that *Spalax* does not develop spontaneous cancer implies that there are changes in other regions of the p53 protein and/or other related proteins to ensure integrity of the related signaling pathways and to maintain homeostasis.

Due to the critical role of p53 in the regulation of apoptosis and DNA repair, we aimed to investigate whether *Spalax* p53 protein sequence shares structural features with other phylogenetically distant mammals that are also adapted to acute-fluctuating hypoxia. Such features may indicate joint adaptations of the cellular stress response via p53 that are related to hypoxic stress resistance. Here, we have identified changes in p53 domains, which are known to include intrinsically disordered regions [41] that are highly diverse in evolution [42], and found that these domains' sequences are similar among mammals adapted to stressful hypoxic conditions, more than expected from phylogeny. These domains harbor binding sites of p53 with proteins that participate in the DDR and in certain metabolic pathways such as replication protein A 70-kDa DNA-binding subunit (RPA70), the histone acetyltransferase p300, and silent information regulator 2 (Sir2) [43–45]. Thus, changes in these domains could modulate the way in which p53 orchestrates these pathways under extreme hypoxic conditions in the subterranean habitat.

## Results

### Different intra-group similarities for hypoxia-tolerant and hypoxia-sensitive taxa

**Fig. 1:**
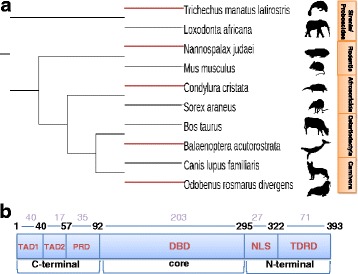
Schematic representation of data used in the study **a** Balanced experimental design. Phylogenetic tree showing the evolutionary relationships between pairs of hypoxia-tolerant and hypoxia-sensitive species. *Red lines* represent hypoxia-tolerant species. The hypoxia tolerant species were paired with their closest hypoxia-sensitive phylogenetic relative, which has an available p53 sequence; **b** Diagram of p53 domains (human p53). P53 domains include: transactivation subdomain 1 (TAD1), transactivation subdomain 2 (TAD2), proline-rich domain (PRD), DNA binding domain (DBD), nuclear localization signal (NLS), and tetramerization and regulatory domains (TD/RD). Domain lengths are in *blue*, amino-acid positions are in *black*, and domain names are in *red*

We hypothesized that certain functional domains in p53 have convergently evolved among hypoxia-tolerant species. If this is true, it is expected, for example, that some domains in *Spalax* p53 are more similar to the corresponding domains of other hypoxia-tolerant (HT) species, such as diving mammals, than to those of hypoxia-sensitive (HS) species, such as murine rodents, although *Spalax* is much closer to murines. To test this, we used a balanced experimental design (Fig. 1a) in which a single representative HT species and a single representative HS species is tested for different taxonomic groups (e.g., carnivores, rodents, even-toed ungulates, insectivores, etc.). In public databases we were able to find five representative HT species whose p53 genes were sequenced, belonging to five different mammalian taxonomic orders. Accordingly, five pairs of closely related species were used, where in each pair one is HT and the other is HS. In this test, amino acid sequence distances were calculated according to the Poisson correction model (see Methods) for the following p53 domains (Fig. 1b): transactivation subdomain 1 (TAD1), transactivation subdomain 2 (TAD2), proline-rich domain (PRD), DNA binding domain (DBD), nuclear localization signal (NLS), and tetramerization and regulatory domains (TD/RD). For each domain in p53, it was then tested whether the pairwise distances within the group involving HT species are smaller than those of the matched group of HS species (H_1_ hypothesis). In addition, the test was repeated using the estimated pairwise divergence time published in the literature [16].

Following the test described in Methods, we calculated Mann–Whitney statistics *U* that characterizes the difference in intra-group similarities (based on all-to-all distances in each group). *P*-values for this statistics were calculated in two ways: using *U* distribution (Mann-Whitney [46]) and distribution obtained by Monte-Carlo simulations (Table 1). Monte-Carlo simulations were used to avoid biases caused by intra-group dependencies derived from the usage of each species more than once when calculating the distances of all possible pairs of species in each group (see Methods). Monte-Carlo simulations were conducted with *k*_*HT*_ = *k*_*HS*_ = 5 and *n*_*HT*_ = *n*_*HS*_ = 5*4/2 = 10, where *k* represents the number of species in each group (five in our test) and *n* represents all possible pairs of species in each group (ten in our test). *U*-statistic for TAD2, DBD, and TD/RD domains was significantly smaller (with FDR adjusted *p* < 0.05), allowing the rejection of the H_0_ hypothesis that components of vectors *S*_*HT*_ and *S*_*HS*_ have the same joint distribution (see Methods). These observed significant differences are unlikely to be explained only by phylogeny, as no significant differences in the intra-group domain similarities were detected using the estimated pairwise divergence time.

**Table 1:** Domains with different intra-group similarities for hypoxia-tolerant (HT) and hypoxia-sensitive (HS) taxa

	Evolutionary tree	TAD1	TAD2	PRD	DBD	NLS	TDRD
*U*_obs_	35	49	12	74	9	50	3
*p*_*MW*_	0.2791	0.4844	0.0015	0.0313	0.0005	0.4861	0.0001
*p*_*MW*_ *-FDR*		0.4861	0.0029	0.0468	0.0015	0.4861	0.0003
*p*_*MC*_	0.413	0.4895	0.0095	0.0768	0.0046	0.4882	0.0005
*p*_*MC*_ *-FDR*		0.4895	0.019	0.1152	0.0140	0.4895	0.0030

Statistics *U* and *p*-values were calculated for domain sequences of p53 and for evolutionary tree positions (see Methods). *P*_*MW*_ for p53 domains corresponds to *p*-value calculated in a standard one-tailed Mann–Whitney test [46]. *P*_MC_ correspond to *p*-values calculated in our extension on *U* test using Monte-Carlo simulations (see Methods). As can be seen it the table, the estimated ‘divergence time’ calculated based on evolutionary tree is unlikely to explain differences between the HT and HS groups, seen in the TAD2, DBD, and TDRD domains

To further verify that the obtained results remain consistent when a wider range of species is used (see Methods), we also compared *Spalax* p53 protein sequence, as a reference sequence to: (1) the entire NCBI *nr* database; (2) p53 sequences of 46 other species, which include all HT species whose p53 sequence was sequenced, as well as additional species that are adapted to other types of metabolic stress (Additional file 1: Table S1, Additional file 2: Figure S1, hypoxic- metabolic- and non-stress groups). The results of these tests (Additional file 3: Table S2) were consistent with the results based on the balanced experimental design for TAD2 and TD/RD (but not DBD), and indicated that these domains in *Spalax* are more similar to those of HT species than to those belonging to species with the highest phylogenetic relatedness to *Spalax*.

### Stress-related patterns in p53 TAD2, TD, and RD

The results above may indicate that unique sequence patterns have convergently evolved in some p53 domains of hypoxia-tolerant species. Accordingly, we investigated candidate sequence patterns in p53 that could specify the similarity between species adapted to stressful hypoxic conditions. Here, “pattern” means the same combination of residues, in the same order, with a constant number of residues between them. We have identified two types of patterns in TAD2 and TD/RD domains of p53: (1) patterns that are more frequent in species from the hypoxic-/metabolic-stress groups relative to species from the non-stress group; (2) patterns that are characteristic solely to all “hypoxic-stress” species (Additional file 1: Table S1) and exist in none of the species included in the metabolic-/non-stress groups.

To identify the first type of patterns, the complete p53 sequences of 47 species from the three above-mentioned groups were aligned by MAFFT (sorted by input order that was determined according to the respective groups). Positions that led to incorrect matching between homologous residues were manually corrected. Common patterns in TAD2 and TD/RD were manually identified and the frequencies of these patterns in the hypoxic-/metabolic-stress groups relative to the non-stress group were subjected to Fisher’s 2 × 2 exact test. In addition, the significance of these patterns in all three groups was tested by Fisher’s 2 × 3 exact test. This data set was used as it represents a wide range of species in terms of stress adaptations, so that (1) we could verify that the patterns identified in p53 protein sequence exist in most or all of hypoxia-tolerant species (2) patterns identified as different between the two extremes (hypoxic vs. non-stress groups), could be further evaluated in mammals experiencing other types of stresses (metabolic stress group). Nevertheless, as this data set is not absolutely phylogeneticaly balanced (see Methods), only the identified patterns for which the *p-value* was smaller than 0.005 were selected and retested in the mentioned balanced experimental design (see Fig. 1a).

**Fig. 2:**
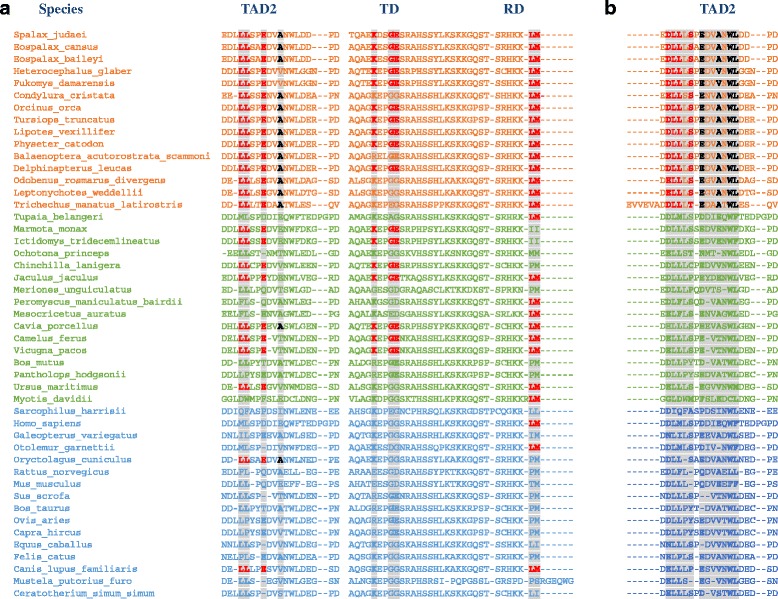
Distribution of positions composing stress-related patterns in p53 TAD2 and TD/RD: **a** Patterns that are more frequent in species included in the “hypoxic” (*red*)/“metabolic” (*green*) “-stress” groups relative to those included in the “non-stress” (*blue*) group. Residues composing the patterns are marked in bold-red (LLXXE in TAD2, KXXGE and LM in TD/RD). An additional pattern was more frequent only for the “hypoxic-stress” species relative to “non-stress species” (LLXXEXXA in TAD2), marked in *bold red and black*; **b** Pattern identified in all “hypoxic-stress” species and in none of the species included in either the “metabolic-” or “non-” “stress” groups. This pattern is a combination of two motifs: [DE]LLX[ST] (*bold red*) and [DE]XX[AV]XWL (*bold black*)

Among the identified patterns (Fig. 2) that were found to be significantly more frequent in species included in the hypoxic-stress group relative to the non-stress group (Additional file 4: Table S3), some of the patterns were also found in the metabolic-stress group, but with a lower level of significance. In TAD2, the pattern LLXXE was significantly more frequent in the hypoxic (*p* < 0.0001) and metabolic (*p* < 0.05) groups compared to the non-stress group. Position A52 (*Spalax* p53; corresponds to E51 in human p53), and its related patterns EXXA and LLXXEXXA, were significantly more frequent only in the hypoxic-stress group (*p* < 0.005 and 0.0001, respectively). In TD/RD, the patterns KXXGE and LM were significantly more frequent in the hypoxic-stress group (*p* < 0.0001 for both patterns) and in the metabolic-stress group (*p* < 0.005 and 0.05, respectively) relative to the non-stress group. The patterns LLXXEXXA, EXXA and LM in TAD2 and RD (but not in TD) were still significantly more frequent in the hypoxic-stress group vs. the non-stress group of the balanced experimental design (*p* < 0.05), despite the small number of species included in each group (Additional file 4: Table S3, Additional file 5: Figure S2).

The next step of our analysis was to check whether the residues participating in the patterns identified in TAD2, TD, and RD (see shaded columns in Fig. 2a) are sufficiently informative to enable the robust classification of species into the three groups: hypoxic-stress, metabolic-stress, and non-stress. We used the “classification trees” algorithm to classify an extended list of mammals (66 rather than the initial 47 species, see Additional file 1: Table S1, Additional file 6: Figure S3), based on the identified positions in p53 as predictors (see Methods). The classification (Additional file 4: Table S3, Additional file 7: Figure S4) showed that all species from the hypoxic-stress group (subterranean and diving mammals) had very high probabilities of being assigned to this group. An analogous result (namely, a classification that corresponds to the subjective assignment) was obtained for the species that belonged to the non-stress group. In the metabolic-stress group, most of the species showed high probability of being assigned to stress-related groups (mostly the metabolic-stress group). Interestingly, in this category, mammals with large body mass tended to have lower probabilities of being classified into stress groups. Nineteen (19) additional species (that are not included in any of the hypoxic/metabolic/non-stress groups, see Methods) were approximately equally distributed between non-stress and stress-related groups. The most important predictor (Additional file 4: Table S3) was found to be p53 position 51 (human p53) in TAD2. These results provide further support that adaptation to stressful hypoxic conditions is predicated by changes at the identified positions.

A second type of pattern in TAD2 (Fig. 2b) domain defines unambiguously (one-to-one) the species included in the hypoxic-stress group. This pattern can be described as a combination of two motifs: [DE]LLX[ST] and [DE]XX[AV]XWL. The combination of these two motifs was found in all hypoxia-tolerant species, but was not found in any of the other species included in the sample (total 47 species). We then checked whether this pattern exists in the 19 additional species (out of the total 66, see Additional file 1: Table S1). The pattern was not found in any of these species, with one exception: the African elephant. In other words, among 66 species tested, this pattern was found only in hypoxia-tolerant species and in the African elephant. Nevertheless, for the African elephant, the pattern in p53-RD (LM) was not found, and thus it is distinguished from the hypoxic-stress group.

In addition, as TAD2 of p53 was shown to resemble TAD2 domain of Krueppel-like factor 1 (KLF1) [47], we also compared the sequences of KLF1-TAD2 domain of hypoxia-tolerant species to those of hypoxia-sensitive species (according to the balanced experimental design). We did not identify any hypoxia-specific pattern (Additional file 5: Figure S2) in KLF1-TAD2. This may indicate that the considered patterns are associated with unique functionality of p53 rather than with a functionality related to a global hypoxic response.

### 3D models of p53 in complex with other DDR proteins provide evidence of a unique DDR in *spalax*

To investigate the possible functional role of the residues identified in p53 as ‘stress-related’ (Fig. 2), we first allocated the available structures of p53 in complex with other proteins in which the interaction includes these residues. We subjected TAD2 and TD/RD sequences to protein BLAST, querying the Protein Data Bank (PDB) database. Only a few structures of the proteins that interact with p53 via these domains have been resolved for human proteins to date (for main examples see Additional file 8: Table S4). We then examined the literature related to these structures, and found that the residues included in the stress-related patterns are indeed directly involved in the interactions of p53 with some of the proteins (Table 2). For example, the pattern ‘EXXA’ (*Spalax* p53) corresponds to residues ‘DXXE’ (positions 48–51, human p53), which are responsible for electrostatic interactions between p53 and other proteins such as RPA70 and p62.

**Table 2:** Residues included in the stress-related patterns participate in the binding of p53 with DDR proteins

p53 interaction with protein (PDBID)/pattern residues sp53 (hp53)	*LL (ML)*	*EXXA (DXXE)*	*LM (LM)*
P300 (2MZD)	L45 binds to nonpolar patches on the surface of Taz2 domain of p300	Salt bridges and hydrogen bonds between D48 and E51 and Taz2 domain of p300	
RPA70 (2B3G)		Electrostatic interactions of D48 and E51 with RPA70N	
CBP (2 L14, 1JSP)	Hydrophobic interactions of M44,L45 with the NCBD domain of CBP		Hydrophobic interactions of L383 and M384 with the bromodomain of CBP
P62 (2RUK)		Electrostatic interaction of D48 and E51 with the PH domain of p62	
Tfb1 (2GS0)		Electrostatic interaction of E51 with Tfb1	
HMGB1 (2LY4)	Hydrophobic interaction of M44,L45 with HMGB1		
Sir2 (1MA3)			Hydrogen bond of L383 with sir2
S100B (1DT7)			Hydrophobic interaction of L383 with S100B

Some of the residues composing the stress-related patterns in TAD2 and RD participate in *Spalax* (s) p53 direct interface with DDR proteins. Specific interactions via these residues between DDR proteins and human (h) p53 are described for each protein. The data for each protein was obtained from manuscripts related to the resolved structures of the protein complex with p53 in humans (PDBID is mentioned in the table for each protein)

**Fig. 3:**
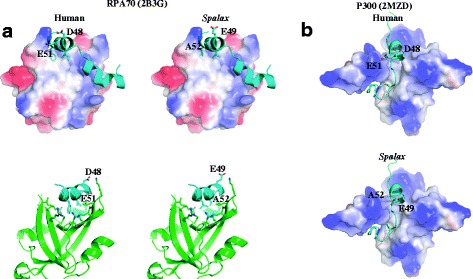
Model of *Spalax* p53 N-terminal (p53N) interaction with: **a** RPA70 repair protein. *Left* – Crystal structure of human p53N fragment (33–60) bound to RPA70N (PDBID 2B3G). The p53 fragment is depicted as a cyan helix and RPA70N as an electrostatic surface (*upper panel*) or as green ribbons (*lower panel*). *Right* – Model of the *Spalax* p53–RPA70 complex, based on the human complex. **b** P300 co-activator. *Upper panel* - The structure of human p53N bound to p300 (PDBID 2MZD). *Lower panel* - Model of the *Spalax* p53–p300 complex, based on the human complex. From this analysis, it seems that the human p53 interface with RPA70 and P300 involves more hydrophobic and ionic interactions than the *Spalax* interface, making the complexes more stable

In addition, we observed large differences between *Spalax* and humans with respect to p53 interactions with proteins involved in DDR pathways via TAD2, as demonstrated in Additional file 9: Figure S5. Therefore, we hypothesized that p53’s interactions with these proteins are different in *Spalax* compared to humans. We then specifically examined p53’s interaction with RPA70 by model replacement of the human p53 residues participating in the direct interface with RPA70, with the corresponding residues in *Spalax*. From this analysis, it seems that the human p53–RPA70 interaction interface involves more hydrophobic and ionic interactions relative to *Spalax* and therefore, the interaction in *Spalax* is less stable (Fig. 3a). Importantly, the residues that mediate the interaction with p53 are identical in humans and *Spalax* in the RPA70 protein (data not shown), unlike the interface-residues in the p53 protein, which are different between the two species. This suggests that p53 and RPA70 did not co-evolve and strengthens the conclusion that the interaction between them is different in *Spalax* compared to humans. Similar results were obtained when we modeled *Spalax*-p53’s interaction with p300 via TAD2. The interface in the p300 side of the interaction was conserved (data not shown); however, we have identified reduced electrostatic interactions in *Spalax* compared to humans due to different interface-residues in p53 (Fig. 3b). We then used MutaBind server to estimate the putative changes in the binding affinity of *Spalax* p53 interactions with other DDR proteins (Additional file 8: Table S4). This analysis supported the possibility that *Spalax*’s stress-related substitutions in TAD2 reduce the binding affinity of p53 to other DDR proteins such as RPA70 and p62, compared to humans. For the stress-related pattern in TD/RD (owned by both *Spalax* and humans) it was demonstrated that this pattern potentially increases the binding affinity of p53 to proteins such as sir2, compared to other species that do not own the pattern.

### Site-specific positive selection in p53

**Fig. 4:**
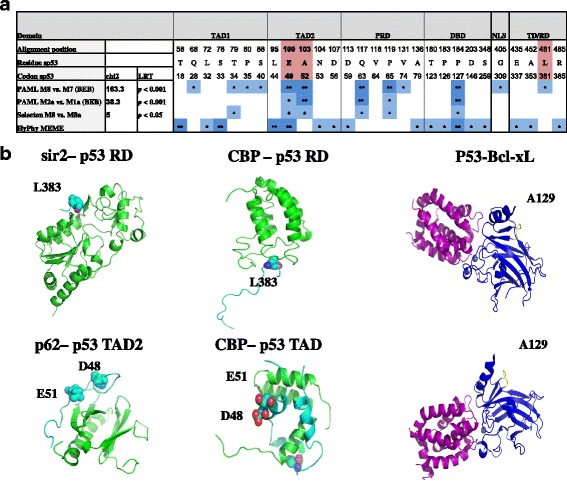
Site-specific positive selection in p53 protein. P53 sequences of 65 mammalian species were analyzed using models implemented by PAML, Selecton, and HyPhy tools (**a**). Significant positively-selected sites are marked with blue and presented on the relevant positions of *Spalax* p53 sequence. (In PAML: probability (omega >1) >0.95* or >0.99**; in Selecton: lower bound of the confidence table >1; in Hyphy: *p*-value ≤ 0.05* or ≤0.01**, proportion ≥ 0.05). Positions composing stress-related patterns E49, A52, L381 in *Spalax* p53 (corresponding to D48, E51 and L383 in human p53, respectively) are marked with pink; **b** Examples of protein-protein interactions presumably affected by changes in positions which compose the stress-related patterns and signals for positive selection (*dN/dS* >1). Left and middle panels - positions in p53 TAD/RD (*cyan*) in complex with CBP, Sir2, and p62 proteins (*green*), as modeled for human p53. Residues D48 and E51 are in balls and are illustrated in p53 interactions with p62 (PDBID 2RUK) and CBP (PDBID 2 L14) via TAD2. Position L383 is illustrated in balls in p53 in complex with Sir2 (PDBID 1MA3) and CBP (PDBID 1JSP) via RD. The indicated positions participate in the binding by mediating hydrophobic and ionic interactions (Table 2). An additional position that was identified as positively selected by all tested models (*right panel, marked in yellow*) in p53 DBD (A129, human p53) is illustrated in the human model (PDBID 2MEJ) of p53 (*blue*) in complex with Bcl-xL (*purple*)

The adaptive role of the sites in which residues composing the above-mentioned stress-related patterns are located was further investigated. We hypothesized that specific p53 amino acid sites in TAD2 and TD/RD were positively selected during evolution. By ‘positive selection’, we mean amino acid substitutions that conferred adaptive advantages under selective pressure. PAML package and Selecton online-server were used to estimate codons’ non-synonymous vs. synonymous substitution rates (*dN/dS*). A likelihood ratio test (LRT) was conducted comparing two nested models: a null model that assumes no positive selection and an alternative model that assumes positive selection. The same 65 sequences were also analyzed using the HyPhy model MEME (Mixed Effects Model of Episodic Diversifying Selection), which can identify sites that were positively selected only in a small fraction of the tree branches. Positions E49, A52 in *Spalax* p53-TAD2 (corresponding to D48, E51 in the human p53) and position L381 in *Spalax* p53-RD (corresponding to L383 in the human p53), which participate in the stress-related patterns, were significantly identified as positively selected by 4, 3, and 1 models out of 4, respectively (see Fig. 4a, Additional file 10: Table S5). The putative role of these positions is illustrated in the 3D structures of p53’s interactions with several proteins via TAD2 and RD (Fig. 4b). Accordingly, since TAD2 seems to have a unique stress-related structure and function, positions E49 and A52 (*Spalax* p53) inside this domain are promising candidates for further analysis. Notably, all tests implemented in Selecton, PAML, and Hyphy identified another residue in the DBD of p53 as a significantly positively selected site: P127 in *Spalax* p53, corresponding to A129 in human p53. This position is located in a loop that fine-tunes the angle between two stapled beta-strands (Fig. 4b). The importance of this position could be related to the role of the two beta-strands’ edges. For example, these edges include positions such as K120 (human p53), which is directly involved in p53 interactions with the DNA and the anti-apoptotic protein Bcl-xL [48].

## Discussion

In this study, we identified similarities in p53 functional domains that are shared between mammals adapted to stressful hypoxic conditions. TAD2 and TD/RD of p53 are rapidly evolving, compared to other domains in p53, such as the DBD [49]. Nevertheless, they are similar between mammals that are not phylogenetically closely related, but share the ability to endure extreme changes in oxygen levels. We suggest that this similarity is an indication of convergent evolution, resulting from selective pressures that induced common molecular adaptations via these p53 domains. We further identified sequence patterns in these domains that are more frequent in mammals adapted to acute-fluctuating hypoxia and demonstrated that the residues participating in the identified patterns can predict species classification according to stress characteristics. An additional pattern in TAD2 was observed in all hypoxia tolerant-species and in none of the other species participating in this study (a total of 66 tested), with one exception: the African elephant. Interestingly, this mammal is cancer-resistant and expresses a unique DNA damage response that is attributed to mechanisms related to p53 [50]. The latter pattern is a combination of two motifs: [DE]LLX[ST], which includes Ser46 and may relate to the binding of proteins that induces its phosphorylation, and [DE]XX[AV]XWL, which includes a motif that is parallel to a motif found in p53-TAD1, which is attributed to the binding of p53 with MDM2 [51] and thus may relate to the competitive binding of MDM2 to p53-TAD2 and p53-TAD1 [52].

Protein sequence motifs, which are located in rapidly evolving intrinsically disordered regions of a hub protein and include segments that mediate its binding with other proteins, fits well with the definition of Short Linear Motifs (SLiMs) [53–55]. These motifs represent a functional change in a flexible low-affinity binding region, which may divert the regulation of a complex signaling network. We suggest that the patterns identified in this study may potentially serve as SLiMs that have evolved among hypoxia-tolerant species. The evolution of SLiMs seems to be very complex and is not yet well understood. Nevertheless, recent theories based on evidence from the past decade [56] suggest that the appearance of SLiMs could be random and “evolutionarily transient” (i.e., *ex nihilo* lost and gained along lineages). This unique evolutionary process may minimize the dependencies between phylogenetically related species and between species and their ancestors; however, it does not contradict events of positive and negative selection along their evolution. In this study, we also showed that positions of residues that make up the identified patterns (E49 and A52 in *Spalax* p53, corresponding to D48 and E51 in human p53), can be considered as positively selected. Importantly, these residues stabilize electrostatic interactions between p53 and proteins such as the cofactor and histone acetyltransferase p300 and the DNA-repair proteins RPA70 and Tfb1/p62 [57–61].

The intrinsically disordered nature of TAD2 and RD enables them to interact with a range of proteins that function as a complex network. Among them are DNA-repair proteins (Additional file 8: Table S4) that when defected cause susceptibility to a variety of congenital cancers and progeroid syndromes (genetic disorders that mimic physiological aging). These syndromes are attributed to the failure of genome-maintenance mechanisms [62], suggesting the involvement of TAD2 and TD/RD in p53-mediated regulation of genomic stability [35]. Only a few 3-D structures of the proteins in complex with p53 via TAD2 and TD/RD have been solved to date, while other interactions are still unknown. Thus, the possible role of residues making up the patterns identified in this study has yet to be elucidated. Nevertheless, to get indications of the possible adaptive importance of the specific changes in these domains in *Spalax* p53, we applied these changes *in silico* in human p53 and showed, albeit indirectly, some evidence for decreased affinity between the modified p53 (according to the hypoxia-tolerant pattern) and RPA70 compared to human p53. This complies with a previous study [63] demonstrating that a few combinations of p53 mutations: [L43A, **M44A**, **L45A**], or [**D48H**, D49H] or [I50A, **E51A**, Q52A], have disrupted the binding of p53 to RPA70. Each of these combinations includes residues that participate in the pattern identified in the current study as stress-related in TAD2 (highlighted in bold). Other studies [64, 65] have demonstrated that the mutations **D48H** and D49H in p53-TAD2 preserved the transactivation function of p53, but disrupted p53 interaction with RPA70 and the subsequent inhibition of homologous recombination (HR) by p53 [65]. This finding suggests the involvement of these residues in the sequestration of RPA70, which is required for HR suppression, and therefore it may be concluded that an inhibition of HR suppression (i.e., HR promotion) might occur in *Spalax*.

TAD2 of p53 functions as a single-stranded (ss) DNA mimic and interacts with proteins that also interact with ssDNA [58, 59]. This DNA mimicking ability may have a role in the dynamics of these proteins’ translocation to the damage site. For example, RPA70 has higher affinity to ssDNA than to p53, and upon DNA damage, it detaches from p53 and integrates with ssDNA at the damage site [58]. Thus, the potential decrease in the binding affinity of p53 to RPA70 in *Spalax* may lead to faster recruitment of RPA70 in the case of DNA damage. Another protein that interacts with p53 via TAD2 and shows similar structural competition with ssDNA is breast cancer 2 protein (BRCA2), which is also involved in double-strand break repair and HR [66, 67]. There is evidence that residues in TAD2, including the above-mentioned D48 and E51 (human p53), play a role in the binding of p53 to BRCA2 [67], implying a similar role in the recruitment of BRCA2 to the damage site. In this interaction, it was suggested that charged residues, such as D48 and E51 (corresponding to E49 and A52 in *Spalax* p53) attract the partner domains of BRCA2 to p53 at the initial phase of binding, whereas hydrophobic residues, such as L43 and L45 (human p53), which is also included in the identified patterns, are associated with the folding of the participating activation domain in BRCA2.

P53 transactivation is regulated by p300, which also affects p53 turnover, depending on the cellular context and the environmental stimuli, e.g., those that induce DNA damage [68]. A recent study investigating the structure of p53-TAD2 in complex with p300 [61] indicates the involvement of almost all the residues identified in the TAD2-stress-related pattern in this interaction. As in the case of RPA70, mutations in positions D48 and E51 (human p53) were shown to induce a decrease in the binding affinity of p53 to p300 [61]. This fits our modeling of the p53-p300 interaction via TAD2 indicating a potentially weaker binding in *Spalax* relative to humans. Importantly, under hypoxia, p53 crosstalks with the hypoxic response transcription factor hypoxia inducible factor 1 (HIF-1) and both compete for p300 as a co-activator to enhance transcription of their target genes [69]. This competition may suggest that the possible decrease in the binding affinity of *Spalax* p53 to p300 indirectly increases p300 availability to HIF-1 and thus enhances HIF-1-mediated transcription (hypoxic response) on the account of p53-mediated-transcription (apoptotic response). This could serve *Spalax* as a complementary mechanism to its constitutively high level of HIF-1 expression [70], as the upregulation of HIF-1 alone is not sufficient to enhance its target-genes transcription, and a compatible amount of available p300 is needed. The potential enhancement of HIF-1-mediated transcription on the account of p53 transcription may shift the balance in *Spalax* cells between survival and apoptosis, in favor of survival.

Most interesting is to examine proteins that interact with both TAD2 and TD/RD of p53, as such interactions can point to a functional link between these two domains. A functional link between TAD2 and RD of p53 was previously demonstrated in the case of the homeodomain-interacting protein kinase-2 (HIPK2), which its knockdown inhibited S46 phosphorylation in TAD2 and K382 acetylation in RD; both S46 phosphorylation and K382 acetylation are required for p53–mediated apoptosis [71]. CREB-binding protein (CBP) belong to the same protein family [72] as p300 and share a similar structure and function as co-activators. Both interact with p53 via TAD2 and acetylate the same residue (K382, human p53-RD) [73, 74]. This residue is also a substrate for the NAD-dependent protein deacetylase SIRT1 (human sirtuin) [75]. Hence, CBP/p300 and SIRT1 may compete for binding p53 in this location in RD. It was shown that the adjacent L383 (human p53), which is included in the stress-related patterns identified in this study, participates in p53 interaction with CBP and SIRT1 via RD, which is required for the acetylation/deacetylation of K382 in p53 (Table 2, Fig. 4). Acetylation of p53 by p300 positively regulates p53 activity, while deacetylation by SIRT1 negatively regulates p53 activity, suppresses apoptosis, and prolongs cellular survival in response to DNA damage [75]. It can be suggested that the patterns we found in p53, may promote p53 deacetylation by SIRT1 on the account of p53 acetylation by p300/CBP, due to the potential decrease in p300/CBP binding to p53 (via TAD2) that may indirectly increase the availability of K382 in RD to SIRT1. SIRT1 homologs have been shown to induce slow-aging in *Caenorhabditis elegans*, *Drosophila*, and mice [76]. It was suggested that this effect is achieved by delaying apoptosis via p53 and giving cells more time to repair the damage [77]. We see the similarities in p53 protein sequence found between *Spalax* and other stress-tolerant and phylogenetically distant mammals as a gateway for investigating potential evolutionary-convergent interconnected mechanisms that may relate its tolerance to extreme changes in oxygen levels to longevity and cancer resistance.

## Conclusions

In this study we showed that the p53 domains TAD2 and TD/RD are more similar between species adapted to hypoxic stress than expected from phylogeny. Patterns shared in these domains among hypoxia-tolerant species include residues that mediate the binding of p53 with critical DDR proteins. Part of the residues are located in positions that were identified as positively selected and potentially play a role in reducing the binding of p53 with p300 and RPA70, suggesting a possible change in p53-mediated regulation of the DDR among species adapted to extreme changes in oxygen levels. Further investigation of TAD2 and TD/RD in the context of hypoxia and DNA repair may lead to progress in understanding cellular hypoxia tolerance, cancer resistance, and aging.

## Methods

### Data set and experimental design

In the current study, p53 sequences of 66 placental mammals were used (Additional file 1: Table S1, Additional file 6: Figure S3). From this group of 66 species, 47 species (Additional file 2: Figure S1) were classified into 3 groups: (1) “hypoxic-stress” group, which includes 15 species adapted to acute and transient environmental hypoxia (e.g., subterranean and diving mammals). Species in this group acquired the ability to inhabit environments that are characterized by extreme and frequent oxygen fluctuations and oxygen deprivation. In this group, we included all known-to-date mammals that, to the best of our knowledge, fit this criterion and have the full length of the p53 protein sequence published. The information regarding mammalian adaptations to different types of hypoxic stresses is available in a review by Ramirez et al. [78], and in our previous study on *Spalax* [4]; (2) “non-stress” group, which includes 16 species that do not endure any type of hypoxic stress (organismal/external/chronic/acute) or other types of stresses affecting cellular metabolism and extreme changes in availability of oxygen to tissues and cells; (3) “metabolic stress” group, which includes 16 species that fit neither the acute-hypoxic nor the non-stress criteria, and are known to endure other types of metabolic stresses such as hibernation, dehydration, extreme temperatures, chronic hypoxia, flying, or different combinations of these stresses (Additional file 1: Table S1). Extra caution should be taken when interpreting the results of the statistical analysis related to this group due to its heterogeneity. The remaining other 19 mammals could not be classified into any of the groups due to insufficient or inconclusive information in the literature or because including them in one of the groups strongly disturb the balance between groups. These mammals were termed as “additional” group and their p53 sequences were mostly used in analyses for which prior classification is not required, such as positive selection.

Species classification into the three first groups specified above was based on the summarized literature regarding each species from the viewpoint of stress adaptations. The classification was optimized as explained below, so that stress adaptations related to p53 could be investigated. For example, some mammals were classified as “non-stress” despite the fact they have semi-aquatic family members (e.g., fishing cat and mink are relatives of cat and ferret, respectively) or evidence of semi-aquatic adaptations (e.g., rhinoceros and humans) [79]. In addition, in this experimental design, the ability to obtain balanced proportions of species from different taxonomic orders and families in each group was also limited by the number of species for which the p53 sequence had been published and by the actual adaptations of the species. Thus, for example, in the metabolic-stress group, there are more rodents than in the other groups. Due to the mentioned limitations, an additional experimental design was used in which each hypoxia-tolerant species was paired with its closest hypoxia-sensitive phylogenetic relative that has an available p53 sequence (see Fig. 1a). Specifically, we selected all pairs of species so that in each pair: (1) there is a unique last common ancestor, namely: not the common ancestor of any other pair of species in the test; (2) one species is hypoxia-tolerant and the other is hypoxia-sensitive. It should be noted that two species from the “additional group” (shrew and elephant) were used in the balanced experimental design to represent hypoxia-sensitive species. This is because these species are the most closely related species (for which p53 sequence was published) to the stare-nosed-mole and manatee, respectively, and despite some stress-related inconclusive data (for example, it is not clear whether shrew faces metabolic stress due to its small body mass).

Protein and mRNA sequences were retrieved from NCBI protein and nucleotide databases, respectively [http://www.ncbi.nlm.nih.gov/]. Habitat and behavioral information were obtained from the Animal Diversity Web (ADW) online database [80]. Taxonomy information was collected from the NCBI Taxonomy browser [81]. Maximal lifespan and adult weight information were obtained from AnAge database [12]. Accession numbers, taxonomy information, lifespan, weight, and additional information related to stress adaptations are available in Additional file 1: Table S1.

### Analysis of the p53 protein sequence and its functional domains

Forty-seven p53 sequences of species included in the hypoxic-, metabolic- and non-stress groups were aligned using MAFFT (multiple alignment using fast Fourier transform) L-INS-i (v7), which applies an iterative refinement method for local pairwise alignment, based on the BLOSUM62 model [82]. To obtain p53 fragments for each functional domain in p53, the alignment result was trimmed using AliView alignment editor [83] to the following domains: TAD1, TAD2, PRD, DBD, NLS, and TD/RD, according to relative positions in human p53 (Fig. 1b). Gaps were removed from the sub-alignments, and the resulting fragments were used for further analysis as described below. The fragments of each p53 functional domain were aligned using MAFFT (as described above). These alignments were used to create a distance matrix according to a Poisson correction model [84], using MEGA6 [85].

### Domain specific intra-group similarity

In order to test whether pairwise distances within a group involving hypoxia-tolerant species and a matched group of hypoxia-sensitive species are similar, the following test was performed. We characterized domain specific intra-group similarity for group *G* of *k* taxa by a vector *S*_*G*_ of *n = k*(*k*-1)/2 distances, scored based on the selected domain for all pairs of taxa from this group. We then tested the following hypothesis H_0_: for the selected domain, components of vectors *S*_*HT*_ and *S*_*HS*_ have the same intra-group joint distribution. Testing this hypothesis by a standard one-tailed Mann–Whitney *U* test is problematic as the components of each of these two vectors are not independent. For example, information on *d*_*ij*_ and *d*_*jk*_ provides some information on *d*_*ik*_. To overcome this difficulty, we extended the standard *U* test by taking into account this type of dependency in row data as described below. Analogously to a standard *U* test, we used test statistic *U* equal to the number of pairs (out of all *n*_*HT*_ *n_*HS*_) of components of vectors *S*_*HT*_ and *S*_*HS*_ such that *S*_*HT*_ > *S*_*HS*_. To conduct the test on real data, we estimate the distribution of *U* values under hypothesis H_0_ by the following Monte-Carlo [86] simulations: In each of *N* = 100,000 runs we simulated *k*_*HT*_ + *k*_*HS*_ random points in Euclidean space. Coordinates of these points were simulated as realization of independent random values with uniform distribution from 0 to 1. For groups of the first *k*_*HT*_ and of the next *k*_*HS*_ points, we calculated all *n*_*HT*_ and *n*_*HS*_ intra-group (dependent) distances as well as *U*-value. We further estimate *p*-value for the observed real vectors *S*_*HT*_ and *S*_*HS*_ as the proportion of simulated runs (out of *N* = 100,000) with *U*-***E*** *U* ≥ *U*_obs_-***E*** *U*, where ***E*** *U* = *(n*_*HT*_ *n_*HS*_ *)*/2 is an expectation of statistic *U* under hypothesis H_0_. If this *p*-value is less than some selected cutoff (e.g., 0.05) then the hypothesis H_0_ should be rejected.

### Comparing *Spalax* p53 domains to the domains of other species

*Spalax* p53 domains were compared to the p53 domains of a range of species: (1) species included in the entire NCBI non-redundant (*nr*) database (2) selected 46 species, as described below. We used two types of similarity measures (i) *Spalax* p53 domains were used as Blastp queries against the *nr* database, which includes most of the non-redundant protein sequences. Blastp hits were then sorted by E-value; (ii) The distances of p53 domains belonging to selected 46 species, from *Spalax* were calculated using Poisson correction model. Fisher’s exact was used to test whether the domain-distance categories (namely, ‘close’ and ‘distant’ to *Spalax*) are significantly associated with the stress-tolerance categories. The categories ‘distant’ and ‘close’ were measured by: (a) expected distance by phylogeny; (b) observed domain sequence distance. An ‘expected’ 2 × 2 contingency table was built with the counts of ‘hypoxia-tolerant’/‘hypoxia-sensitive’ species that are ‘close’/‘distant’ from *Spalax*, where ‘close’ species were classified as those belonging to the *Rodentia* or *Lagomorpha* clades (with less than 87 million years divergence time from *Spalax*). In this test, ‘hypoxia-tolerant’ refers to 15 species included in the hypoxic-stress group and ‘hypoxia-sensitive’ refers to 32 species included in the metabolic- and non-stress groups (see Additional file 1: Table S1). The ‘observed’ 2 × 2 contingency table included the same total counts of ‘distant’/‘close’ species as in the ‘expected’ table, but the distance measure was domain distance rather than phylogenetic distance. Fisher’s exact test was conducted using the R function ‘fisher.test’. The data set tested included a higher proportion of species categorized as phylogenetically “distant” than phylogenetically “close” among the HT group (15 species), and a lower proportion for the HS group (32 species). Thus, Fisher’s exact test odds ratio (OR) parameter was adjusted and defined as (H_close_/H_dist_)/(S_close_/S_dist_); where: H_close_ and H_dist_ are the counts of HT species that are either closely-related or distantly-related to *Spalax*, respectively; S_close_ and S_dist_ are the counts of HS species that are either closely related or distantly related to *Spalax*, respectively. The parameter ‘*or’* (odds ratio) of the R function ‘fisher.test’ was calculated from the ‘expected’ table, to reflect the expected proportions of HT species in the ‘close’ vs. ‘distant’ group (see Additional file 3: Table S2, TAD2 example). In addition, the test was repeated for a subset of 43 species and of 41 representative species (for groups of species separated by <10 million years, only a single species was selected, and the rest were excluded). Furthermore, the test was repeated 100 times with random sub-samples of 38 and 33 species, out of the 43 species (jackknife resampling method) and the mean *p*-value was calculated. In the above-described test we used a data set that included all known hypoxia-tolerant species for which p53 sequence was published. This comprises species within clades that consist of only hypoxia-tolerant species (Additional file 2: Figure S1), and thus it may introduce potential biases due to dependent samples. Therefore, the results were tested also in a balanced data set (as explained in the previous Methods sections).

### Classification analysis

For classification analysis, we used the data-mining method “Boosting Trees for Classification” (http://www.statsoft.com/Textbook/Boosting-Trees-Regression-Classification) [87]. This method predicts the membership of objects in different groups based on the values of one or more predictor variables. The p53 protein sequences of 66 species (see Additional file 1: Table S1) were aligned using CLUSTALW2 Omega (1.2.1) [88], where each alignment position was considered as a potential predictor variable in the analysis. The actual predictors were set to the positions that were found to be more frequent in species adapted to hypoxic/metabolic stress (see Results, Fig. 2a). The species’ classification into three categories (hypoxic-, metabolic-, and non-stress) was set to be the dependent variable, and the algorithm classified the species based on the defined predictors. The “Training” data was set to be the 47 species that we subjectively assigned to the “hypoxic-stress”, “metabolic-stress”, and “non-stress” groups. This classification was determined according to information we collected from the literature related to the ability of the species to endure different types of hypoxic/metabolic stresses (see Additional file 1: Table S1). Thus, these species were used by the boosted-tree-algorithm to build the model, and their subjective classification was set to be the initial observed values. The remaining 19 species (“Additional” group) comprised the “Test” data that was used to evaluate the fitting of the model over successive iterations and did not participate in the building of the model. The initial observed values of these species were tentatively set to the intermediate stress type, namely: “metabolic stress”.

### Structural analysis of *Spalax* p53

PyMOL Molecular Graphics System, Version 1.5.0.4 Schrödinger, LLC was used for molecular visualization of proteins’ interaction with p53. For structures involving p53 TAD2 (Additional file 9: Figure S5), all residues that differ between human and *Spalax* p53 sequences were marked and displayed in balls. For visualizing ‘Selecton’ positive-selection results (Fig. 4), the residues in TAD2 and RD for which *dN/dS* >1 were marked by the Selecton software in the relevant PDB file and then displayed by PyMOL, accordingly. For modeling the structure of *Spalax* p53 in complex with RPA70 according to the human complex, only the residues participating in the direct interface between p53 and RPA70 were replaced by *Spalax* residues and displayed. For modeling the structure of *Spalax* p53 in complex with p300, only residues that differ between *Spalax* and humans and participate in the stress-related patterns were replaced and displayed. PDB files were retrieved from the PDB database [89]. MutaBind Server [90] was used to estimate the putative changes in the binding affinity of *Spalax* p53 to other DDR proteins, compared to other species.

### Site-specific positive-selection analysis

A phylogenetic tree of 65 species was built according to the phylogenetic tree topologies in TimeTree of Life [16], a resource for estimations of divergence-time between species. Then, the tree-branch lengths were optimized using Rate4Site [91] according to the p53 protein sequences of the 65 species, aligned by MAFFT (Additional file 11: Table S6). Since site-specific positive-selection analysis tools are extremely sensitive to alignment errors, when aligning p53 codon sequences, we manually inspected, using AliView alignment editor [83], the results of two codon-alignment methods: (1) PAGAN v.0.47 [92] and (2) the method implemented by the Selecton server [93], which is based on MUSCLE multiple alignment [94]. Due to an ambiguous result for amino acid residues in the disordered TAD2 when using PAGAN alignment, the aligned codon sequences produced by Selecton were chosen for use as input data in the analytical procedures described below, in addition to the phylogenetic tree of 65 species for which the branches were optimized.

Positive-selection analysis was conducted using: (1) The HyPhy module MEME [95]. This method reports site-specific *dN/dS* (i.e., non-synonymous vs. synonymous substitution rate), allowing the estimated *dN/dS* rate to change between both sites and branches, as expected in the case of p53, where it is likely that at different sites, only some branches were positively selected. For this analysis, following the HyPhy manual: SLAC analysis was run first, followed by MEME analysis, both analyses with HyPhy QuickSelectionDetection.bf module; (2) Maximum likelihood estimation of site-specific *dN/dS* using CODEML program in PAML version 4.8 [96, 97]. This program allows comparing the models M1a vs. M2a, and M7 vs. M8. The null-models M1a and M7 estimate the probabilities of 2 and 10 classes of *dN/dS* for each site, all with *dN/dS* between 0 and 1. The alternative models M2a and M8 include an additional site class with *dN/dS* >1 estimate. Using a likelihood ratio test (LRT), *p*-values were calculated from χ^2^ distribution. If a specific alternative model was found to be significant (LRT *p*-value < 0.05), then positive selection was tested for each site (*p* > 0.95 for the *dN/dS* >1 site class). Site class probability was tested using Bayes Empirical Bayes (BEB) [98]. (3) The Selecton online server [93] [http://selecton.tau.ac.il/]. This server implements an empirical Bayesian method for *dN/dS* calculation and allows to compare the M8 model (that assumes selection) vs. the M8a null model (that assumes no selection) by LRT as described above, and to identify site-specific selection, accordingly. It is important to clarify that site-specific positive selection models do not provide information regarding the lineage in which the positive selection might occur. Methods that provide information regarding the site and the branch (e.g., branch-site model, PAML) were not used since these methods are not reliable for regions containing alignment gaps, which were tested here [99].

## Additional files

Additional file 1: Table S1. Additional information on the species employed in the study. (XLS 56 kb)
Additional file 2: Figure S1. Phylogenetic tree of 47 species. (PDF 15 kb)
Additional file 3: Table S2. P53 sequences distances. (XLS 90 kb)
Additional file 4: Table S3. Stress related patterns and classification analysis. (XLS 83 kb)
Additional file 5: Figure S2. Stress related patterns in p53-TAD2 and KLF1-TAD2. (PDF 23 kb)
Additional file 6: Figure S3. Phylogenetic tree of 66 species. (PDF 16 kb)
Additional file 7: Figure S4. Classification analysis. (PDF 4479 kb)
Additional file 8: Table S4. Critical DDR proteins that interact with TAD2 and/or TD/RD. (XLS 42 kb)
Additional file 9: Figure S5. Differences between human and *Spalax* in p53-TAD2 interactions. (PDF 1461 kb)
Additional file 10: Table S5. Positive selection results. (XLS 443 kb)
Additional file 11: Table S6. Positive selection input. (XLS 269 kb)
