# Supplementary material for: Adaptive patterns in the p53 protein sequence of the hypoxia- and cancer-tolerant blind mole rat Spalax
Source: BMC Evol Biol. 2016 Sep 2;16:177. doi: 10.1186/s12862-016-0743-8 (PMC5010716; doi:10.1186/s12862-016-0743-8)
Supplement: Additional file 2: Figure S1. — Phylogenetic tree of 47 species. (PDF 15 kb) [file 12862_2016_743_MOESM2_ESM.pdf]

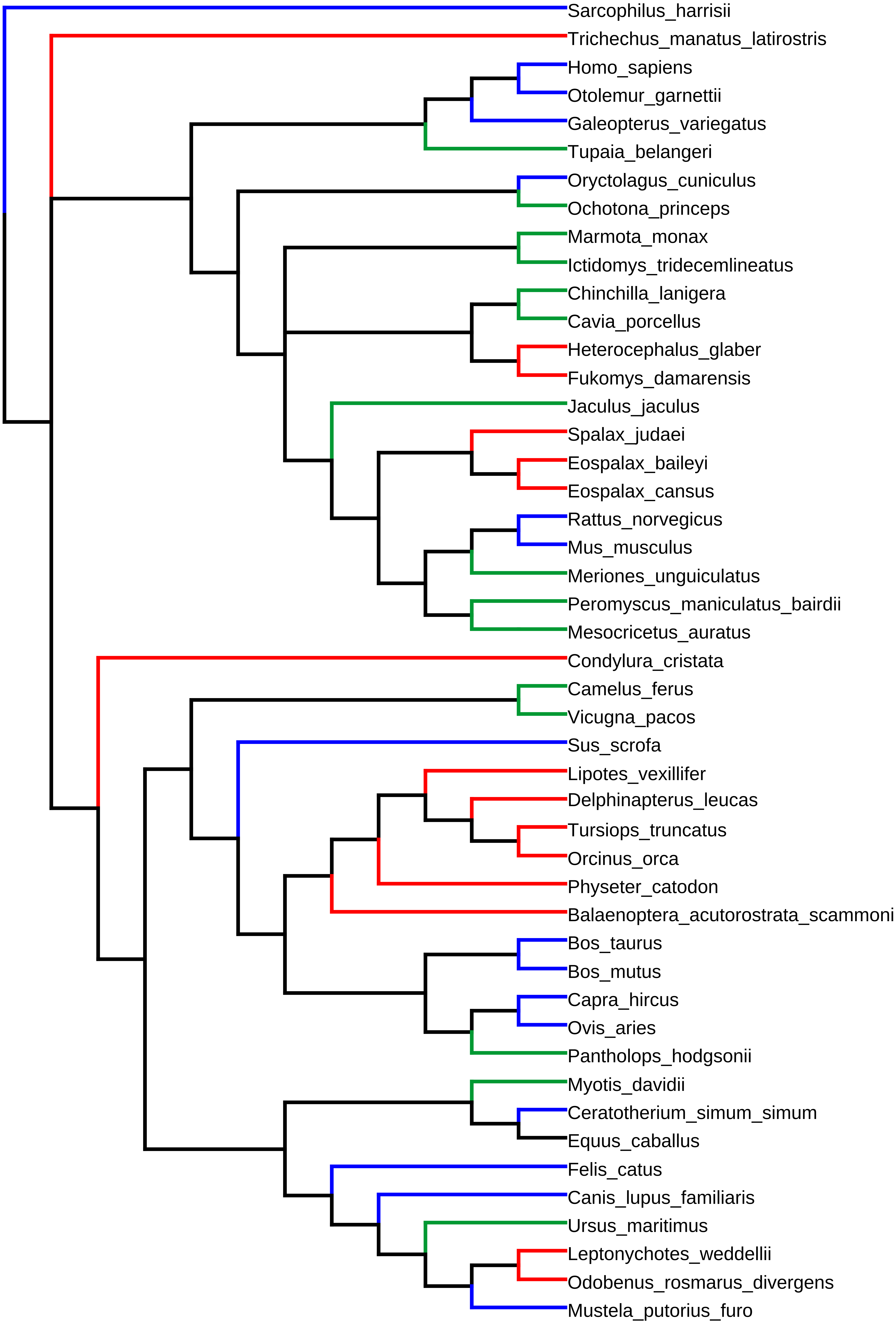

Sarcophilus_harrisii
Trichechus_manatus_latirostris
Homo_sapiens
Otolemur_garnettii
Galeopterus_variegatus
Tupaia_belangeri
Oryctolagus_cuniculus
Ochotona_princeps
Marmota_monax
Ictidomys_tridecemlineatus
Chinchilla_lanigera
Cavia_porcellus
Heterocephalus_glaber
Fukomys_damarensis
Jaculus_jaculus
Spalax_judaei
Eospalax_baileyi
Eospalax_cansus
Rattus_norvegicus
Mus_musculus
Meriones_unguiculatus
Peromyscus_maniculatus_bairdii
Mesocricetus_auratus
Condylura_cristata
Camelus_ferus
Vicugna_pacos
Sus_scrofa
Lipotes_vexillifer
Delphinapterus_leucas
Tursiops_truncatus
Orcinus_orca
Physeter_catodon
Balaenoptera_acutorostrata_scammoni
Bos_taurus
Bos_mutus
Capra_hircus
Ovis_aries
Pantholops_hodgsonii
Myotis_davidii
Ceratotherium_simum_simum
Equus_caballus
Felis_catus
Canis_lupus_familiaris
Ursus_maritimus
Leptonychotes_weddellii
Odobenus_rosmarus_divergens
Mustela_putorius_furo

Fig. S1. **Phylogenetic tree of 47 species.**

The phylogenetic tree was built according to estimated time of species divergence, obtained from the TimeTree website (book and online database). Line colors represent stress categories (hypoxic-stress in red, metabolic-stress in green, and non-stress in blue).
