# Supplementary material for: Adaptive patterns in the p53 protein sequence of the hypoxia- and cancer-tolerant blind mole rat Spalax
Source: BMC Evol Biol. 2016 Sep 2;16:177. doi: 10.1186/s12862-016-0743-8 (PMC5010716; doi:10.1186/s12862-016-0743-8)
Supplement: Additional file 5: Figure S2. — Stress related patterns in p53-TAD2 and KLF1-TAD2. (PDF 23 kb) [file 12862_2016_743_MOESM5_ESM.pdf]

		P53-TAD2	p53-RD
**Rodentia**	Spalax_judaei	EDL**LL**SP**E**DV**A**NWLDD-	SRHKK**LM**
**Cetartiodactyla**	Balaenoptera_ac	DDL**LL**SP**E**DV**A**NWLDE-	SRHKK**LM**
**Carnivora**	Odobenus_rosmar	DE-**LL**LS**E**GV**A**NWLDA-	SRHKK**LM**
**Insectivora**	Condylura_crist	EE-**LL**SS**E**NV**A**NWLDE-	SHHKK**LM**
**Sirenia**	Trichechus_mana	DD-**LL**LT**E**DA**A**TWLES-	SRHKK**LM**
**Rodentia**	Mus_musculus	DDL**LL**PQDVEE-FFEG-	SRHKKTM
**Cetartiodactyla**	Bos_taurus	DD-**LL**PYTDV**A**TWLDE-	SCHKKPM
**Carnivora**	Canis_lupus_fam	DE-**LL**LP**E**SVVNWLDE-	SRHKK**LM**
**Insectivora**	Sorex_araneus	DDL**LL**SP-DVENWLDK-	SRHKKPM
**Proboscidea**	Loxodonta_afric	DD-**LL**LS**E**DT**A**NWLES-	SRHKKPM

		KLF1-TAD2
**Rodentia**	Spalax_judaei	ERYPTCAWDPNVFLANF
**Cetartiodactyla**	Balaenoptera_ac	ERDAATPWDLDLLLTNF
**Carnivora**	Odobenus_rosmar	ERDSAAAWDLDLLLTNF
**Insectivora**	Condylura_crist	ERDEASAWDLDLFLTNF
**Sirenia**	Trichechus_mana	QRDEAVAWDLDLLLTNF
**Rodentia**	Mus_musculus	ERDVTCAWDPDLFLTNF
**Cetartiodactyla**	Bos_taurus	ERDETVAWDLDLLLTNF
**Carnivora**	Canis_lupus_fam	ERDSATAWDLDLLLTNF
**Insectivora**	Sorex_araneus	ERDTVSAWDLDLLLTNF
**Proboscidea**	Loxodonta_afric	ERDETVAWDLDLLLTNF

Fig. S2. **TAD2 of KLF1 does not show hypoxia-specific patterns**

p53-TAD2, p53-RD, and KLF1-TAD2 sequence alignments of species included in the paired experimental design. Species that are hypoxia tolerant are colored in red, species that are hypoxia sensitive are colored in blue, and stress-related patterns in p53 are marked in bold red letters.
