# Supplementary material for: Adaptive patterns in the p53 protein sequence of the hypoxia- and cancer-tolerant blind mole rat Spalax
Source: BMC Evol Biol. 2016 Sep 2;16:177. doi: 10.1186/s12862-016-0743-8 (PMC5010716; doi:10.1186/s12862-016-0743-8)
Supplement: Additional file 6: Figure S3. — Phylogenetic tree of 66 species. (PDF 16 kb) [file 12862_2016_743_MOESM6_ESM.pdf]

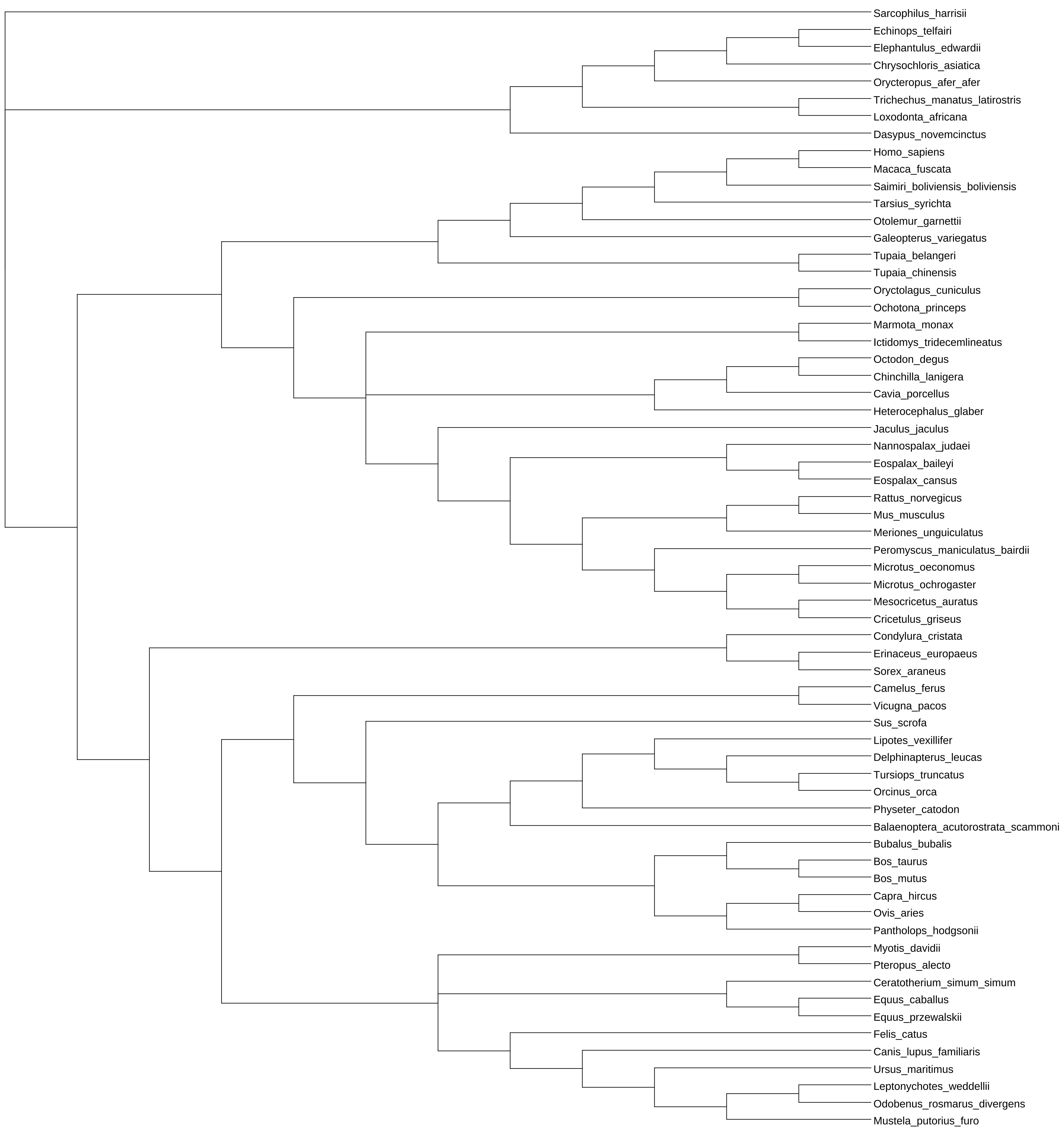
Sarcophilus_harrisii
Echinops_telfairi
Elephantulus_edwardii
Chrysochloris_asiatica
Orycteropus_afer_afer
Trichechus_manatus_latirostris
Loxodonta_africana
Dasypus_novemcinctus
Homo_sapiens
Macaca_fuscata
Saimiri_boliviensis_boliviensis
Tarsius_syrichta
Otolemur_garnettii
Galeopterus_variegatus
Tupaia_belangeri
Tupaia_chinensis
Oryctolagus_cuniculus
Ochotona_princeps
Marmota_monax
Ictidomys_tridecemlineatus
Octodon_degus
Chinchilla_lanigera
Cavia_porcellus
Heterocephalus_glaber
Jaculus_jaculus
Nannospalax_judaei
Eospalax_baileyi
Eospalax_cansus
Rattus_norvegicus
Mus_musculus
Meriones_unguiculatus
Peromyscus_maniculatus_bairdii
Microtus_oeconomus
Microtus_ochrogaster
Mesocricetus_auratus
Cricetulus_griseus
Condylura_cristata
Erinaceus_europaeus
Sorex_araneus
Camelus_ferus
Vicugna_pacos
Sus_scrofa
Lipotes_vexillifer
Delphinapterus_leucas
Tursiops_truncatus
Orcinus_orca
Physeter_catodon
Balaenoptera_acutorostrata_scammoni
Bubalus_bubalis
Bos_taurus
Bos_mutus
Capra_hircus
Ovis_aries
Pantholops_hodgsonii
Myotis_davidii
Pteropus_alecto
Ceratotherium_simum_simum
Equus_caballus
Equus_przewalskii
Felis_catus
Canis_lupus_familiaris
Ursus_maritimus
Leptonychotes_weddellii
Odobenus_rosmarus_divergens
Mustela_putorius_furo

Fig. S3. Phylogenetic tree of 66 species used in this study

Phylogenetic tree was built according to the topologies in TimeTree of Life, a resource for estimations divergence-time between species

Fig. S3. Phylogenetic tree of 66 species used in this study

Phylogenetic tree was built according to the topologies in TimeTree of Life, a resource for estimations divergence-time between species
