# Supplementary material for: Adaptive patterns in the p53 protein sequence of the hypoxia- and cancer-tolerant blind mole rat Spalax
Source: BMC Evol Biol. 2016 Sep 2;16:177. doi: 10.1186/s12862-016-0743-8 (PMC5010716; doi:10.1186/s12862-016-0743-8)
Supplement: Additional file 7: Figure S4. — Classification analysis. (PDF 4479 kb) [file 12862_2016_743_MOESM7_ESM.pdf]

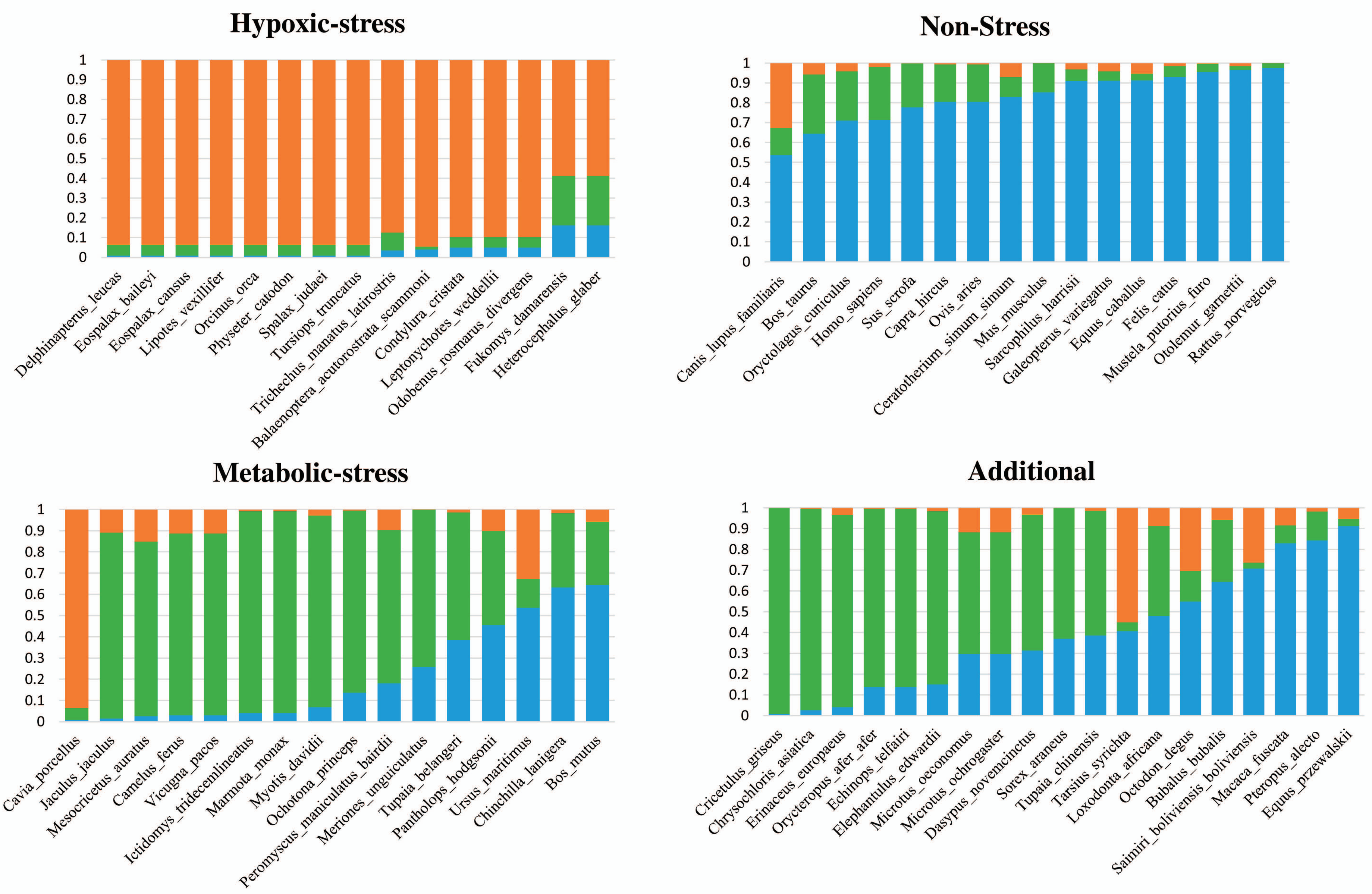
Hypoxic-stress
1
0.9
0.8
0.7
0.6
0.5
0.4
0.3
0.2
0.1
0
Delphinapterus_leucas
Eospalax_baileyi
Eospalax_cansus
Lipotes_vexillifer
Orcinus_orca
Physeter_catodon
Spalax_judaei
Tursiops_truncatus
Trichechus_manatus_latirostris
Balaenoptera_acutorostrata_scammoni
Condylura_cristata
Leptonychotes_weddellii
Odobenus_rosmarus_divergens
Fukomys_damarensis
Heterocephalus_glaber
Non-Stress
1
0.9
0.8
0.7
0.6
0.5
0.4
0.3
0.2
0.1
0
Canis_lupus_familiaris
Bos_taurus
Oryctolagus_cuniculus
Homo_sapiens
Sus_scrofa
Capra_hircus
Ovis_aries
Ceratotherium_simum_simum
Mus_musculus
Sarcophilus_harrisii
Galeopterus_variegatus
Equus_caballus
Felis_catus
Mustela_putorius_furo
Otolemur_garnettii
Rattus_norvegicus
Metabolic-stress
1
0.9
0.8
0.7
0.6
0.5
0.4
0.3
0.2
0.1
0
Cavia_porcellus
Jaculus_jaculus
Mesocricetus_auratus
Camelus_ferus
Vicugna_pacos
Ictidomys_tridecemlineatus
Marmota_monax
Myotis_davidii
Ochotona_princeps
Peromyscus_maniculatus_bairdii
Meriones_unguiculatus
Tupaia_belangeri
Pantholops_hodgsonii
Ursus_maritimus
Chinchilla_lanigera
Bos_mutus
Additional
1
0.9
0.8
0.7
0.6
0.5
0.4
0.3
0.2
0.1
0
Cricetulus_griseus
Chrysochloris_asiatica
Erinaceus_europaeus
Orycteropus_afer_afer
Echinops_telfairi
Elephantulus_edwardii
Microtus_oeconomus
Microtus_ochrogaster
Dasypus_novemcinctus
Sorex_araneus
Tupaia_chinensis
Tarsius_syrichta
Loxodonta_africana
Octodon_degus
Bubalus_bubalis
Saimiri_boliviensis_boliviensis
Macaca_fuscata
Pteropus_alecto
Equus_przewalskii

Fig. S4. Classification analysis of 66 p53 sequences based on positions in p53 TAD2 and TD/RD. The classification analysis result is presented for the 66 species in relation to subjectively predefined groups, according to literature summarized from the viewpoint of stress adaptations. The "classification tree" algorithm was used to reclassify the 66 species based on positions which include residues that were identified as more frequent in stress-related mammals (predictors). Bars represent the predicted probabilities for each species to be in the hypoxic-stress (red), metabolic-stress (green), and non-stress (blue) groups.
