# Supplementary material for: Adaptive patterns in the p53 protein sequence of the hypoxia- and cancer-tolerant blind mole rat Spalax
Source: BMC Evol Biol. 2016 Sep 2;16:177. doi: 10.1186/s12862-016-0743-8 (PMC5010716; doi:10.1186/s12862-016-0743-8)
Supplement: Additional file 9: Figure S5. — Differences between human and Spalax in p53-TAD2 interactions. (PDF 1461 kb) [file 12862_2016_743_MOESM9_ESM.pdf]

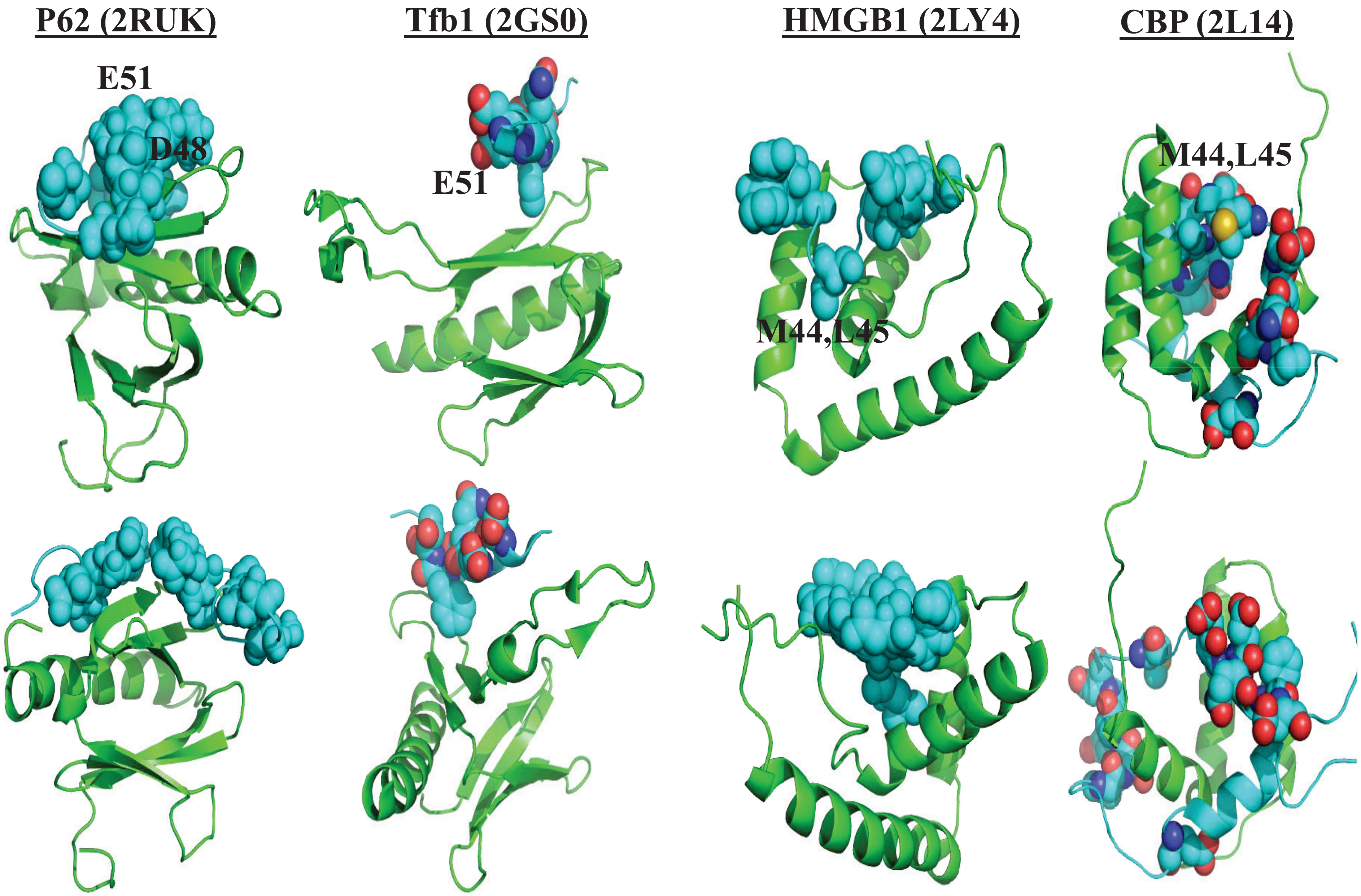

P62 (2RUK)
Tfb1 (2GS0)
HMGB1 (2LY4)
CBP (2L14)
E51
D48
E51
M44,L45
M44,L45

Figure S5. Human p53–TAD2 in complexes with other DNA-damage response proteins.

The presented complexes involve TAD2 of p53. The p53 residues that differ between human and *Spalax* are shown as balls. The p53 fragment is in cyan and fragments of other proteins are in green. Each interaction is presented in two perspectives (shown in upper and lower panels). PDBIDs are mentioned in brackets. Residues participating in the stress-related patterns are marked (e.g., residues D48 and E51 of phosphorylated p53-TAD2 interact with residues in the PH domain of p62; residue E51 is also involved in electrostatic interactions between p53-TAD2 and the p62-yeast homolog, Tfb1; the hydrophobic residues M44 and L45 bind to the top of the hydrophobic groove in the NCBD domain of CBP, and to the concave DNA-binding face of HMGB1).
